# Optimization of Ionic Liquid Based Simultaneous Ultrasonic- and Microwave-Assisted Extraction of Rutin and Quercetin from Leaves of Velvetleaf (*Abutilon theophrasti*) by Response Surface Methodology

**DOI:** 10.1155/2014/283024

**Published:** 2014-08-27

**Authors:** Chunjian Zhao, Zhicheng Lu, Chunying Li, Xin He, Zhao Li, Kunming Shi, Lei Yang, Yujie Fu, Yuangang Zu

**Affiliations:** ^1^College of Resources and Environmental Sciences, China Agricultural University, Beijing 100193, China; ^2^Key Laboratory of Forest Plant Ecology, Ministry of Education, Northeast Forestry University, Harbin 150040, China

## Abstract

An ionic liquids based simultaneous ultrasonic and microwave assisted extraction (ILs-UMAE) method has been proposed for the extraction of rutin (RU), quercetin (QU), from velvetleaf leaves. The influential parameters of the ILs-UMAE were optimized by the single factor and the central composite design (CCD) experiments. A 2.00 M 1-butyl-3-methylimidazolium bromide ([C4mim]Br) was used as the experimental ionic liquid, extraction temperature 60°C, extraction time 12 min, liquid-solid ratio 32 mL/g, microwave power of 534 W, and a fixed ultrasonic power of 50 W. Compared to conventional heating reflux extraction (HRE), the RU and QU extraction yields obtained by ILs-UMAE were, respectively, 5.49 mg/g and 0.27 mg/g, which increased, respectively, 2.01-fold and 2.34-fold with the recoveries that were in the range of 97.62–102.36% for RU and 97.33–102.21% for QU with RSDs lower than 3.2% under the optimized UMAE conditions. In addition, the shorter extraction time was used in ILs-UMAE, compared with HRE. Therefore, ILs-UMAE was a rapid and an efficient method for the extraction of RU and QU from the leaves of velvetleaf.

## 1. Introduction

Velvetleaf (*Abutilon theophrasti* Medik) is one of the main members in Malvaceae and it is a major annual weed in cropland [1]. There are lots of biological activity components in leaves and seeds from velvetleaf [2, 3]. However, there is less information about velvetleaf flavonoids components [4, 5]. It was reported that there were flavonoids in velvetleaf and the main three components were rutin (RU), quercetin (QU), and kaempferol (KA) [4, 6]. Our preliminary study showed that there were rich RU and QU, but a little KA from Chinese velvetleaf.

**Figure 1:**
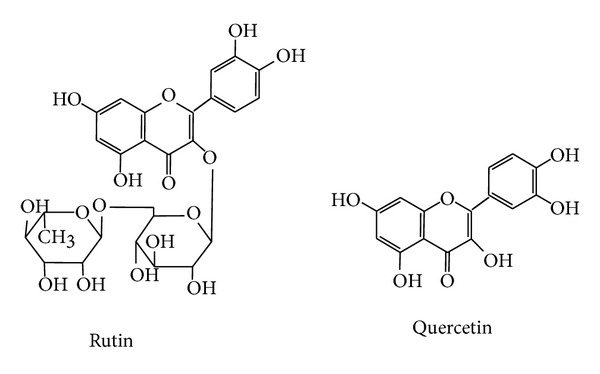
Chemical structures of rutin (RU) and quercetin (QU).

RU (3,4,5,7-tetrahydroxyflavone-3-d-rutinoside) and QU (3,3′,4′,5,7-pentahydroxyflavone), whose structures are shown in Figure 1, are two effective compounds for curing hypertension, diabetes, and cardiac and cerebral vascular diseases [7–9]. In addition, they display the activities of antioxidant [10–12], anti-inflammatory [13, 14], antimicrobial [15], antitumor [16], and antiasthma [17]. In virtue of the above important activities of RU and QU and their plentiful content in velvetleaf, therefore, it is very important to develop a method for the extraction and determination of RU and QU from velvetleaf in order to utilize the abundant velvetleaf resources in China.

Conventional heating reflux extraction (HRE) and ultrasonic extraction (UE) were once applied in the extraction of flavonoids [18]. However, these extraction processes are connected with long extraction time and unsatisfactory recovery. Thus, it is desirable to develop a rapid and efficient extraction method to improve the limitations of conventional extraction of flavonoids [19].

Ionic liquids (ILs) are a kind of salts that display an amazingly lower melting temperature than the boiling point of water and they are often liquid at room temperature. ILs constituted of relatively large asymmetric organic cations and smaller inorganic or organic anions [20]. Due to their negligible vapor pressure, good thermal sensibility, low or virtually no volatility, good miscibility with water and organic solvents, and extractability for various organic compounds, they have recently been widely applied in the extraction and separation from natural product [21–23]. For example, different types of compounds such as essential oils, flavonoids, suberin, polyphenolic compounds, and alkaloids were all extracted by many ILs-based extraction technologies [24–26].

In recent times, microwave-assisted extraction (MAE) has been rapidly developed and proposed as a prospective and influential technique to replace conventional extraction techniques in the extraction of bioactive constituents from plant materials because of its special heating mechanism, moderate capital cost, rapid extraction, and excellent performance [27, 28]. It has been reported that microwave energy can be efficiently absorbed under the ILs as solvents and cosolvents conditions [29]. Considering that ILs can efficiently absorb microwave energy, it is rather an interesting challenge to use ILs as solvent for the MAE of various biomolecules from solid samples. Comparing with conventional organic solvents, ILs are green solvents because their vapour pressure was so lower that ILs are very difficult to evaporate into the environment. In some cases, they could even be well recycled. They can effectively improve the selectivity and the extraction efficiency of the being investigated compounds from plant samples [29].

Ultrasonic is one of the most industrially used methods to enhance the extraction effects due to its mass transfer phenomena [30–32]. Recently, simultaneous ultrasonic/microwave assisted extraction (UMAE) coupled the advantage of microwave and ultrasonic, presenting many advantages [33, 34]. However, there are no reports on ILs-based simultaneous ultrasonic and microwave assisted extraction (ILs-UMAE) of RU and QU from leaves of velvetleaf.

In the present study, a fast and efficient method of ILs-UMAE separation and determination of two major flavones (RU and QU) from leaves of velvetleaf was developed and the effects of extraction time, temperature, ionic liquids concentration, solid-liquid ratio, and microwave power on RU and QU yields were investigated and further optimized by a central composite design (CCD) and response surface methodology (RSM).

## 2. Experimental

### 2.1. Chemicals and Reagents

RU and QU standards were bought from J & K Chemical Ltd. (Beijing, China). All ionic liquids ([C2mim]Br, 1-ethyl-3-methylimidazolium bromide; [C4mim]Br, 1-butyl-3-methylimidazolium bromide; [C6mim]Br, 1-hexyl-3-methylimidazolium bromide; [C8mim]Br, 1-octyl-3-methylimidazolium bromide; [C4mim]Cl, [C4mim]NO3, [C4mim]HSO4, [C4mim]BF4, [C4mim], that is 1-octyl-3-methylimidazolium bromide) were purchased from J & K Chemical Ltd. (Beijing, China). Deionized water for HPLC was purified using a Milli-Q Water Purification System (Millipore, MA, USA). Other analytical reagents were purchased from the Tianjin Kermel Chemical Reagent Co. Ltd. (Tianjin, China).

### 2.2. Materials

The leaves of velvetleaf were collected in autumn from Shuyang County, Jiangsu, China. Voucher specimens were deposited in the herbarium of our laboratory. The materials were dried in the shade at room temperature, powdered by a disintegrator (HX-200A, Yongkang Hardware and Medical Instrument Plant, China), and passed through a stainless steel sieve (40–60 mesh) and stored in closed desiccators at 4°C until use.

### 2.3. Apparatus

Simultaneous ultrasonic and microwave extracting apparatus (CW-2000, Shanghai Xintuo analytical instrument technology Co. Ltd., China, the maximum power of 700 W and a fixed ultrasonic power of 50 W) and ultrasonic extraction device (A KQ-250DB, Kunshan, China) with a maximum power of 250 W were used for the extraction of targets compounds. The HPLC system consisted of a Waters 717 automatic sample handling system series HPLC system equipped with a 1525 pump, a 717 automatic column temperature control box, and a 2487 UV-detector (Waters, USA) that was used for the determination of targets compounds. Chromatographic separation was performed on a HiQ sil-C18 reversed-phase column (4.6 mm × 250 mm, 5 m, KYA TECH).

### 2.4. Extraction Methods

#### 2.4.1. Heating Reflux Extraction (HRE)

A 1.0 g of dried sample powders was put into a round-bottomed flask by adding 20 mL of methanol or 2 M [C4mim]Br; then the flask was placed into oil bath with a reflux device, followed by extracting at 6 h.

#### 2.4.2. Ultrasonic-Assisted Extraction (UAE)

A 1.0 g of dried sample powders was put into a conical flask by adding 20 mL of methanol or 2 M [C4mim]Br; then the conical flask was placed into the ultrasonic extraction device, followed by sonication for 1 h at room temperature.

#### 2.4.3. Microwave-Assisted Extraction (MAE)

A 1.0 g of dried sample powders was put into a special round-bottomed flask by adding 20 mL of methanol or 2 M [C4mim]Br; then the round-bottomed flask was placed into the pressure self-control microwave decomposition system followed by microwave irradiation.

#### 2.4.4. Ultrasonic- and Microwave-Assisted Extraction (IL-UMAE)

**Figure 2:**
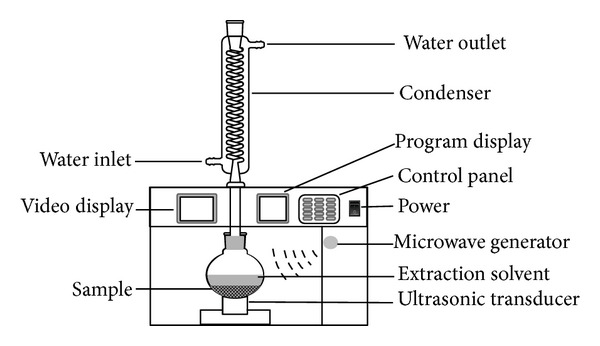
Schematic representation of the UMAE device.

UMAE device was used to separate RU and QU from the leaves of velvetleaf. The apparatus is shown schematically in Figure 2. With cold water running through the condenser of the UMAE system, sample was mixed with methanol or different concentrations of IL solutions, and then the suspensions were irradiated under microwave heating and a fixed ultrasonic power of 50 W. After each irradiation, the obtained extracts were cooled to 25°C, then diluted to 50 mL with water, and filtrated through a 0.45-*μ*m filter for subsequent HPLC analysis.

### 2.5. Determination of RU and QU by HPLC

The diluted extracts were directly injected into the system which was a Waters 717 automatic sample handling system series HPLC system. The conditions of HPLC analysis were as follows: the mobile phase was methanol-acetonitrile-water (40 : 15 : 45, v/v/v) adding 1.0% acetic acid. This mobile phase was filtered through a 0.45 *μ*m membrane filter and then deaerated ultrasonically prior to use. The injection volume was 10 *μ*L and the column temperature was set at 25°C. The flow rate was 1 mL/min. The UV detection wavelength applied was 360 nm. Peak areas of RU and QU were used for quantification with external standard method.

The extraction yield of target analyte was determined as follows:

(1) Yield  (mg/g)  =mean  mass  of  target  analytes  in  sample (mg)mass  of  samples (g).

The mean mass of target analytes in samples was calculated after 3 repeated determinations under the optimized conditions.

### 2.6. Experimental Design

First, the influencing factors of ILs-UMAE, namely, extraction time, temperature, ionic liquids concentration, solid-liquid ratio, and microwave power on the yields of RU and QU were investigated. On the above single factor experiments, the three dominating parameters, that is, microwave power, extraction time, and liquid-solid ratio on the yields of RU and QU, were optimized by RSM. In detail, the effects of three independent variables including extraction time (X1: 6–14 min), liquid-solid ratio (X2: 20–40 mL/g), and microwave power (X3: 300–700 W) at five levels (–1.68, –1, 0, +1, +1.68) were investigated using a central composite design (CCD) with RSM (Table 1).

A total of 20 experiments consisting of 8 factorial points, 6 axial points, and 6 replicates at the central points were performed. Experimental data collected from the designed experiment were analyzed by a response surface regression model using the following second-order polynomial:

(2) $$\begin{array}{c} Y=\beta_{0}+\overset{3}{\underset{i=1}{\sum }}\beta_{i}X_{i}+\overset{3}{\underset{i=1}{\sum }}\beta_{ii}X_{i}^{2}+\overset{2}{\underset{\begin{array}{c} i=1\\ i<j \end{array}}{\sum }}\overset{3}{\underset{j=2}{\sum }}\beta_{ij}X_{i}X_{j}, \end{array}$$

where *Y* represented the response variable; *β*_0_, *β*_*i*_, *β*_*ii*_, and *β*_*ij*_ were the regression coefficients of variables for intercept, linearity, square, and interaction terms, respectively; *X*_*i*_ and *X*_*j*_ were the independent coded variables influencing the response variable *Y*; and *k* represents the number of variables.

### 2.7. Statistical Analysis

Design Expert (DE) software (Trial version 7.0.0, STAT-EASE Inc., Minneapolis, MN, USA) was used to analyze the experimental data and to find the response surfaces of the response models and it was used to decide and assess the statistical significance of the equations. The lack of fit and coefficient of determination (*R* ^2^) were used to evaluate the adequacy of model. The Fisher test value (*F*-value) and their interactions were estimated by the analysis of variance (ANOVA). Finally, in order to decide the adequacy of the fitted model, the actual and predicted values were compared.

The optimum condition for three variables (extraction time: 6–14 min, liquid-solid ratio: 20–40 mL/g, and microwave power: 300–700 W) was acquired by statistical analysis (DE software) [35].

## 3. Results and Discussion

### 3.1. Selection of ILs for UMAE

#### 3.1.1. Effect of Anion

**Figure 3:**
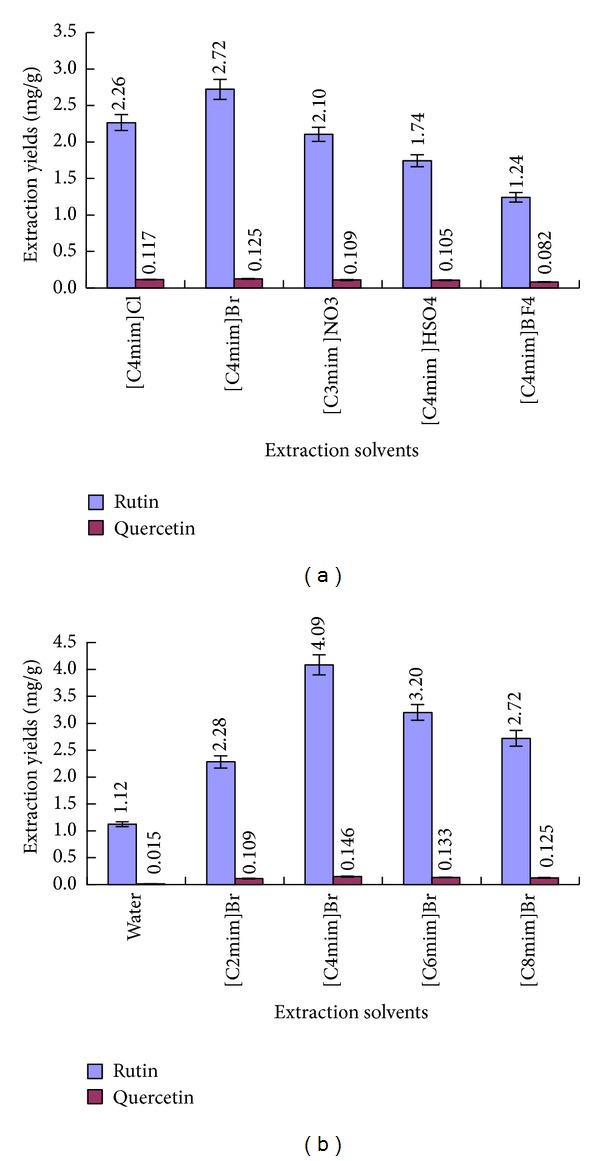
Effect of ionic liquids anions (a) and cations (b) on the extraction yields of RU and QU. Error bars indicate standard deviation (*n* = 3).

The anion identity is an important factor to impact the properties of ILs [36]. Thus, the 1-butyl-3-methylimidazolium ILs with five kinds of different anions (Br^−^, Cl^−^, NO_3_^−^, HSO_4_^−^, and BF_4_^−^) were selected in UMAE. As shown in Figure 3(a), compared with the extraction results using five different types of 1 M ILs solutions with the same cations but different anions, it was apparently found that [C4mim]Br was more efficient than others.

It was probably due to the stronger multi-interactions including *π*-*π*, ionic/charge - charge, and hydrogen bonding between the ILs ([C4MIM]Br) and flavonoids [33].

#### 3.1.2. Effect of the Alkyl Chain Length

The alkyl chain length of the imsidazolium ring of ILs has a significant influence on their physical and chemical properties [37, 38]; thus, the alkyl chain length of ILs will consequently influence the extraction yields of the analytes. In order to investigate the effect of 1-alkyl-3-methylimidazolium-type ILs, ILs with different alkyl chain lengths of cation on the extraction yields of RU and QU were studied in UMAE process. The four kinds of ILs were investigated in UMAE, that is, [C2mim]Br, 1-ethyl-3-methylimidazolium bromide; [C4mim]Br, 1-butyl-3-methylimidazolium bromide; [C6mim]Br, 1-hexyl-3-methylimidazolium bromide; [C8mim]Br, 1-octyl-3-methylimidazolium bromide. Water and different types of 1 M ILs solution were used for the extraction solvents for assessing the extraction yield of RU and QU in UMAE procedure. The results are shown in Figure 3(b), ILs with different alkyl chain lengths of cation significantly influenced the extraction yields of target compounds, and obviously, while [C4mim]Br was used as extraction solvent, the higher extraction yields of RU and QU were obtained than the other three ILs. Compared with the other ILs solutions, water as the solvent, the extraction yields of RU and QU were the most poor. The possible reason was related to the solubility of flavonoids compounds in extraction solvent. The addition of ILs solution improved the extraction yields of target compounds; it may be the reason that the strong dissolvable ability of ILs on target compounds [39–41]. When, the alkyl chain length was more than 4 carbons, the extraction yield of RU and QU distinctly decreased in ILs-UMAE process. Therefore, [C4mim]Br was selected for the further experiments.

### 3.2. Optimization of IL-UMAE Conditions

#### 3.2.1. Single Factor Experiments

**Figure 4:**
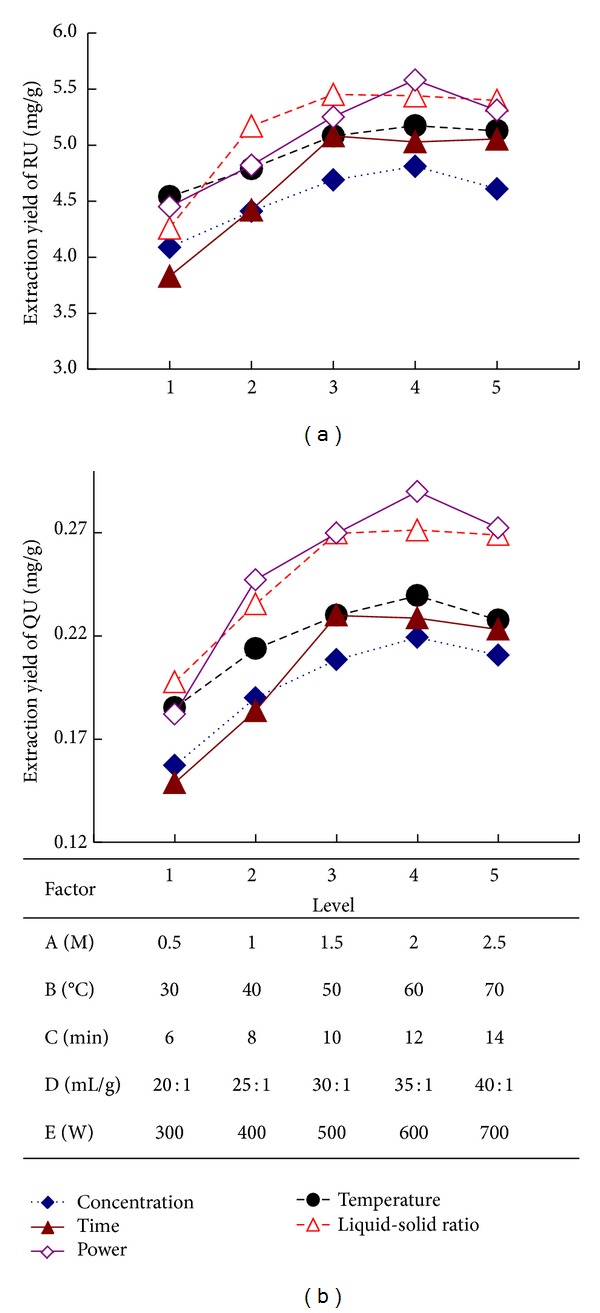
The effect of the concentrations of [C4mim]Br (A), extraction temperature (B), extraction time (C), liquid-solid ratio (D), and microwave power (E) on the extraction yields of RU and QU.

Single factor experiment was performed by one factor varied with different levels, while other factors were fixed. There are many factors affecting the extraction yields of target compounds, which involved the concentration of [C4mim]Br solution, extraction time, liquid-solid ratio, extraction temperature, and microwave power. All results of single factor experiments were shown in Figure 4.

In Figure 4, it can be apparently observed that when the concentration of [C4mim]Br solution was 2 M, the extraction yield of RU and QU was the highest. While the concentration of [C4mim]Br solution was less than 2 M, the extraction yields of target compounds increased rapidly, which might be the reason, with the increasing of [C4mim]Br, both the solubility and the extracting capacity of the solvent were enhanced. At the same time, the capabilities of microwave absorption and microwave conversion were both increased. However, when the concentration of [C4mim]Br solution was more than 2 M, the extraction yields declined. The major cause was that the greater the [C4mim]Br concentration can severely influence the viscosity and the diffusion capacity of solutions. So 2 M of [C4mim]Br was selected for further experiments.

The extraction time is another crucial factor that should be studied to increase the extraction yields of RU and QU. As shown in Figure 4, when the extraction time increased from 6 to 10 min, the extraction yields of the two target compounds increased dramatically. When the time variable was changed from 10 min to 14 min, the extraction yields of the two target compounds reduced slightly. Therefore, 10 min was selected for further experiments.

As for liquid-solid ratio, Figure 4 shows that the extraction yield of two target compounds increased significantly when the liquid-solid ratio increased from 20 : 1 to 30 : 1 mL/g. In certain range, raising the liquid-solid ratio can make sample completely immerse into solvent and increase the mass transfer, and it results in the higher extraction yields of target compounds. Furthermore, with the increase of liquid-solid ratio from 30 : 1 to 40 : 1 mL/g, the extraction yields of RU and QU no longer increased. Hence, 30 : 1 mL/g of liquid-solid ratio was selected for further experiments.

As shown in Figure 4, it can be seen that the extraction yields of RU and QU increased with the increase of the extracting temperature from 30–60°C, which might be because the increasing of the extracting temperature contributes to reducing the viscosity of ILs and enhancing the spread ability and solubility of ILs, which was beneficial to dissolve and extract target compounds. However, when the temperature was higher than 60°C, the extraction yield of RU and QU decreased slightly. The main reason may be the decomposition of some RU and QU at high temperature. Thus 60°C of temperature was selected for further experiments.

It can be shown from Figure 4 that with the increase of microwave power from 300 to 600 W, the extraction yields of RU and QU increased rapidly. When the microwave power was higher than 600 W, the extraction yields of RU and QU began to gradually decrease. Therefore, 600 W of power was selected for further experiments.

#### 3.2.2. Statistical Analysis

The extraction time, liquid-solid ratio, and microwave power were selected as three independent factors, and the dependent variable (response, yields of RU and QU of each run of the experimental design) were investigated by RSM. In order to minimize the effects of the uncontrolled factors, the experimental sequence was randomized (see Table 1). The experimental results obtained at each point are shown in Table 2. These values of the significance of each experimental variable can be justified, which were made of the model fitted, the software generated model coefficients, *R* ^2^-values, Fit-values (*F*-values), and significant probabilities. The response and variables were mutually fitted by multiple regressions. Regression analysis is the general approach to fit the empirical model with the collected response variable data [42]. The second-order polynomial model was generated to describe the empirical relationship between the yields of target compounds and operational conditions (*X*_1_: extraction time, *X*_2_: liquid-solid ratio, and *X*_3_: microwave power) in terms of coded values:

(3) Y1=5.32+0.58X1+0.16X2+0.34X3−0.058X1X2 +0.029X1X3−0.1X2X3−0.48X12−0.24X22−0.27X32,Y2=0.26+0.029X1+0.016X2+8.322×10−3X3 −6.436×10−3X1X2+2.864×10−3X1X3 −2.561X2X3−0.026X12−0.024X22−0.010X32.

**Figure 5:**
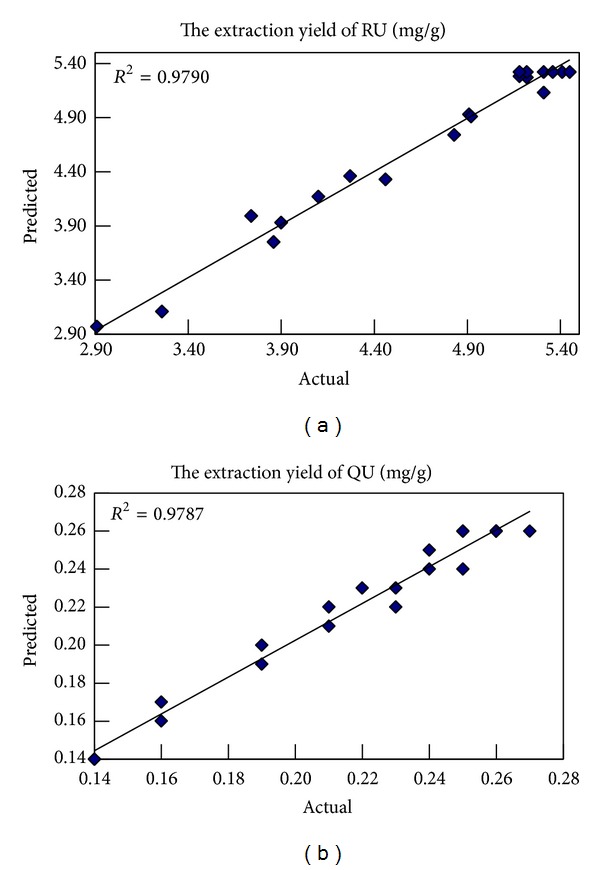
Actual versus predicted values obtained from estimated model.

The predicted values for extraction yield of RU and QU obtained using above model were seen in Table 2. The plot of actual values versus predicted values for the estimated model is shown Figure 5. The relationship between the actual and predicted values showed that the plotted points cluster around the diagonal line. Predicted *R* ^2^ are 0.9790 for extraction yield of RU and 0.9787 for extraction yield of QU, respectively; residual standard deviation (RSD) is 3.34% for extraction yield of RU and 3.92% for extraction yield of QU, respectively.

Positive sign in model of each term represents synergistic effect, while antagonistic effect is represented by negative sign. Analysis of variance (ANOVA) was then used to assess the goodness of fit. The significant quadratic models and the corresponding significant model term for all responses are tabulated in Table 3 for the extraction yield of RU and Table 4 for the extraction yield of QU, respectively.

In Table 3, the model *F*-value of 51.71 with a very low *P* value (*P* < 0.0001) displayed that the generated model was statistically significant and indicated that the extraction yields of RU by IL-UMAE could be well described with this model. It was also observed that the linear term of extraction time (*X*_1_) and microwave power (*X*_3_) have large significant effect on the yield of RU because of the high *F*-value of 191.7 and 65.53, respectively. The quadratic term of extraction time (*X*_1_^2^) and microwave power (*X*_3_^2^) are also significant with *F*-value of 143.34 and 43.04, respectively. According to the software analysis, the lack of fit *F*-value was 3.33, and the *P* value (0.1065) was greater than 0.05 indicating that the lack of fit was not significant relative to the pure error [43].

From Table 4, the model *F*-value of 51.13 with a very low *P* value (*P* < 0.0001) implies that the model is significant. It was clearly observed that extraction time (*X*_1_) and liquid-solid ratio (*X*_2_) have large significant effect on the yield of QU due to the high *F*-value of 158.99 and 45.26, respectively. The quadratic term of extraction time (*X*_1_^2^) and microwave power (*X*_2_^2^) have also significant with *F*-value of 135.98 and 116.53, respectively. According to the software analysis, the lack of fit *F*-value was 2.48, and the *P* value (0.1711) was greater than 0.05 indicating that the lack of fit was not significant relative to the pure error.

### 3.3. Optimization Analysis

**Figure 6:**
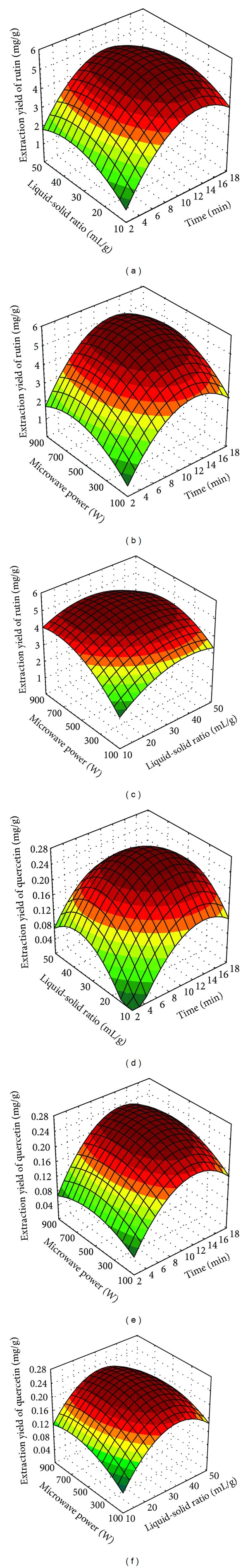
Response surface plots for extracting RU and QU by UMAE: (a) liquid-solid ratio and extraction time, (b) irradiation power and time, (c) irradiation power and liquid-solid ratio on yield of RU, (d) liquid-solid ratio and extraction time, (e) irradiation power and time, and (f) irradiation power and liquid-solid ratio on yield of QU.

The interaction between the variables was shown in Figure 6 and it can be seen from Figure 6 that the 3D response surfaces show that, at high levels of microwave power and an extraction time at a constant ratio of plant material to solvent, the extraction yield was maximal. The optimum extraction conditions (independent variables) proposed by DE software were as follows: extraction time of 12.27 min, liquid-solid ratio of 31.78 mL/g, and microwave power of 533.87 W. The predicted extraction yield under the above conditions was computed as 5.58 mg RU and 0.27 mg QU per 1 g of dried plant material. Considering the precision of extraction device, the optimal condition for extracting RU and QU was selected as extraction time 12 min, liquid-solid ratio 32 mL/g, and microwave power of 534 W. Under the above optimized condition, sample was repeatedly extracted for 6 times, the extraction yields were 5.67 ± 0.12 mg/g for RU and 0.29 ± 0.01 mg/g for QU (the extraction yield values corresponded to the 100% yield values); sample was extracted once, the extraction yields were 5.49 ± 0.16 mg/g for RU and 0.27 ± 0.01 mg/g for QU, and the yields of RU and QU were, respectively, 96.8% and 94.2%.

### 3.4. Comparison of Different Extraction Procedures

**Figure 7:**
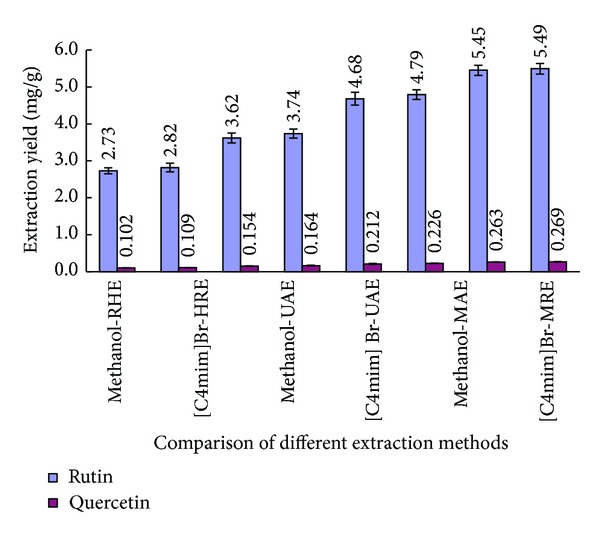
Effect of different extraction methods on yield of RU and QU. Error bars indicate standard deviation (*n* = 3).

A comparison between UMAE and the HRE, UAE, and MAE was performed and the results were seen in Figure 7.

Figure 7 shows that, for any method, it is worth noting that [C4mim]Br and methanol were almost the same as the extraction yields of two target compounds. However, methanol was volatile, flammable, and harmful to human and environment. Therefore, [C4mim]Br was chosen as extraction solvent in this study.

It can be seen from that the extraction yield of RU and QU obtained by ILs-UMAE was, respectively, 5.49 mg/g for RU and 0.27 mg/g for QU, which increased, respectively, 2.01-fold and 2.34-fold compared to conventional methanol-HRE. In conclusion, compared with other three extraction methods, ILs-UMAE had the highest extraction yield of RU and QU from the leaves of velvetleaf with the shortest extracting time. For UMAE, cavitation and microwave irradiation resulted in high effective temperatures and pressures at the interphase between solvent and solid matrix; moreover, microwaves raise the temperature suddenly and disrupt the structure of vegetal cell [34].

### 3.5. Quantitative Analysis by HPLC

**Figure 8:**
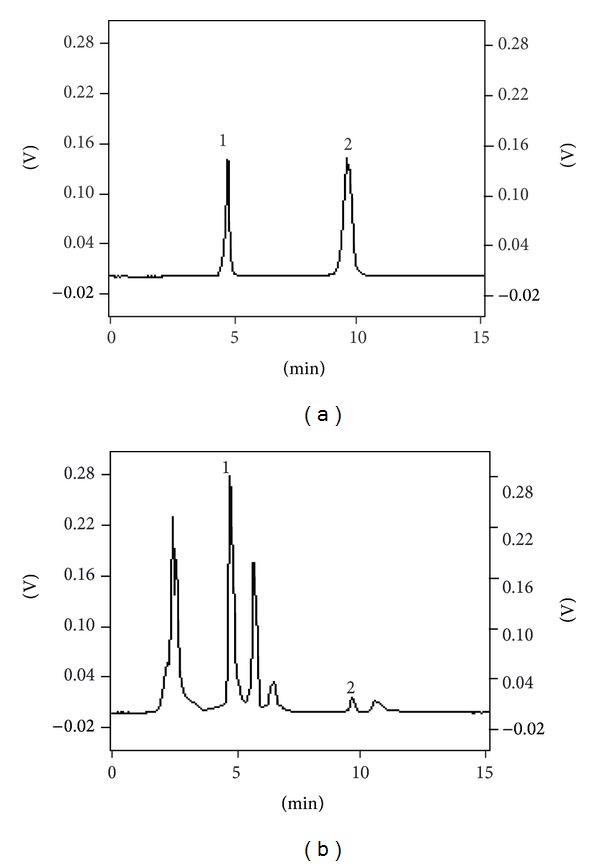
Chromatograms of RU and QU standard mixture (a), sample (b), Peak 1 for RU, and Peak 2 for QU under optimal conditions.

Under the chromatographic conditions of 2.5, the peaks of RU and QU were observed with an acceptable resolution from the peaks of neighboring compounds. The HPLC chromatograms of the analyzed extracts are shown in Figure 8. Furthermore, recoveries were evaluated by standard-addition method, and the extracts were spiked with known quantities of standards. Results showed that the recoveries were in the range of 97.62–102.36% for the RU and 97.33–102.21% for QU with RSDs lower than 3.2% under the optimized UMAE conditions. The reproducibility and recovery proved that the present method was credible.

## 4. Conclusion

In the present study, the ILs-UMAE was used to extract the two objective compounds (RU and QU) from leaves of velvetleaf. According to the single factors experiments and CCD test, we concluded the optimized extraction solvent 2.00 M [C_4_mim]Br, extraction temperature 60°C, extraction time 12 min, liquid-solid ratio 32 mL/g, microwave power of 534 W, and a fixed ultrasonic power of 50 W. The RU and QU extraction yields obtained by ILs-UMAE were, respectively, 5.49 ± 0.16 mg/g and 0.27 ± 0.01 mg/g, which increased, respectively, 2.01-fold and 2.34-fold compared to conventional HRE. In addition to the higher extraction yields, the shorter extraction time was used in ILs-UMAE, which suggested that the IL-UMAE was a rapid and highly effective extraction method for the extraction of RU and QU from the leaves of velvetleaf. Therefore, considering the unique properties of ILs, the IL-UMAE method shows a great promising prospect to be developed as an environmental friendly, rapid, and efficient technique in the extraction of RU and QU from leaves of velvetleafand it can be a promising technique for the extraction of natural active compounds from the other plant.

## Figures and Tables

**Table 1:** Central composite design (CCD) of independent variables for process optimization.

Run	Coded			Actual		
	Factor A (*X*_1_)	Factor B (*X*_2_)	Factor C (*X*_3_)	Time, min (*X*_1_)	Liquid-solid ratio, mL/g (*X*_2_)	Microwave power, W (*X*_3_)
1	1	−1	1	14	20	700
2	−1	−1	1	6	20	700
3	0	0	0	10	30	500
4	−1	1	1	6	40	700
5	0	0	1.68	10	30	836.36
6	1.68	0	0	16.73	30	500
7	1	1	1	14	40	700
8	0	0	0	10	30	500
9	−1	−1	−1	6	20	300
10	1	1	−1	14	40	300
11	0	0	0	10	30	500
12	0	0	−1.68	10	30	163.64
13	−1	1	−1	6	40	300
14	1	−1	−1	14	20	300
15	0	0	0	10	30	500
16	0	0	0	10	30	500
17	0	−1.68	0	10	13.18	500
18	0	0	0	10	30	500
19	0	1.68	0	10	46.82	500
20	−1.68	0	0	3.27	30	500

**Table 2:** Actual and predicted results for the extraction yields of RU and QU.

Run	Actual values		Predicted values	
	The extraction yield of RU (mg/g)	The extraction yield of QU (mg/g)	The extraction yield of RU (mg/g)	The extraction yield of QU (mg/g)
1	5.22	0.23	5.27	0.23
2	3.90	0.16	3.93	0.16
3	5.31	0.26	5.32	0.26
4	4.10	0.19	4.17	0.20
5	5.31	0.25	5.13	0.24
6	4.91	0.24	4.93	0.24
7	5.18	0.24	5.28	0.25
8	5.41	0.26	5.32	0.26
9	3.26	0.14	3.11	0.14
10	4.83	0.22	4.74	0.23
11	5.45	0.27	5.32	0.26
12	3.74	0.21	3.99	0.22
13	3.86	0.19	3.75	0.19
14	4.46	0.21	4.33	0.21
15	5.22	0.25	5.32	0.26
16	5.18	0.26	5.32	0.26
17	4.27	0.16	4.36	0.17
18	5.36	0.25	5.32	0.26
19	4.92	0.23	4.91	0.22
20	2.91	0.14	2.97	0.14

**Table 3:** ANOVA of the fitted quadratic polynomial model for the extraction yield of RU.

Source	Sum of squares	Degrees of freedom (df)	Mean squares	*F* value	*P* value	Remarks
Model	11.20	9	1.24	51.71	<0.0001	Significant
*X*_1_	4.61	1	4.61	191.70	<0.0001	Significant
*X*_2_	0.36	1	0.36	15.05	0.0031	
*X*_3_	1.58	1	1.58	65.53	<0.0001	Significant
*X*_1_ *X*_2_	0.027	1	0.027	1.13	0.3129	
*X*_1_ *X*_3_	0.0068	1	0.0068	0.28	0.6071	
*X*_2_ *X*_3_	0.081	1	0.081	3.38	0.0959	
*X*_1_^2^	3.38	1	3.38	140.34	<0.0001	Significant
*X*_2_^2^	0.85	1	0.85	35.40	0.0001	
*X*_3_^2^	1.04	1	1.04	43.04	<0.0001	Significant
Residual	0.24	10	0.024			
Lack of fit	0.19	5	0.037	3.33	0.1065	Not significant
Pure error	0.056	5	0.011			

Cor. total	11.44	19				

**Table 4:** ANOVA of the fitted quadratic polynomial model for the extraction yield of QU.

Source	Sum of squares	Degrees of freedom (df)	Mean squares	*F* value	*P* value	Remarks
Model	0.034	9	0.037	51.13	<0.0001	Significant
*X*_1_	0.012	1	0.012	158.99	<0.0001	Significant
*X*_2_	3.312 × 10^−3^	1	3.312 × 10^−3^	45.26	<0.0001	Significant
*X*_3_	9.458 × 10^−4^	1	9.458 × 10^−4^	12.92	0.0049	
*X*_1_ *X*_2_	3.314 × 10^−4^	1	3.314 × 10^−4^	4.53	0.0592	
*X*_1_ *X*_3_	6.561 × 10^−5^	1	6.561 × 10^−5^	0.90	0.3660	
*X*_2_ *X*_3_	5.246 × 10^−5^	1	5.246 × 10^−5^	0.72	0.4170	
*X*_1_^2^	9.950 × 10^−3^	1	9.950 × 10^−3^	135.98	<0.0001	Significant
*X*_2_^2^	8.527 × 10^−3^	1	8.527 × 10^−3^	116.53	<0.0001	Significant
*X*_3_^2^	1.588 × 10^−3^	1	1.588 × 10^−3^	21.70	0.0009	
Residual	7.317 × 10^−4^	10	7.317 × 10^−5^			
Lack of fit	5.213 × 10^−4^	5	1.043 × 10^−4^	2.48	0.1711	Not significant
Pure error	2.105 × 10^−4^	5	4.209 × 10^−5^			

Cor. total	0.034	19				
